# Impact of oil massage on newborn behavioural responses in rural India

**DOI:** 10.6026/973206300200160

**Published:** 2024-02-29

**Authors:** B Mahalakshmi, Patel Urvi Sureshbhai, Amita Shilpa Gottlieb, N Sivasubramanian, Gnanadesigan Ekambaram, Ravi kant

**Affiliations:** 1 Department of Paediatric Nursing, Nootan College of Nursing, Sankalchand Patel University, Visnagar, Gujarat - 384315, India; 2 Department of obstetric and gynaecological Nursing, Graphic Era College of Nursing, Graphic Era Deemed to be University, Dehradun, Uttarakhand -248002, India; 3 Department of Psychiatric Nursing, Nootan College of Nursing, Sankalchand Patel University, Visnagar, Gujarat - 384315; India; 4 Department of Physiology, Nootan Medical College & Research Centre, Sankalchand Patel University, Visnagar, Gujarat, India; 5 Department of Microbiology, Nootan Medical College & Research Centre, Sankalchand Patel University, Visnagar, Gujarat, India

**Keywords:** Newborns, oil massage, behavioural responses, rural-setting

## Abstract

The initial weeks of a newborn's life are marked by rapid physiological and behavioural adjustments as the infant adapts to the external environment. This critical period necessitates attentive care, prompting exploration into traditional practices such as oil massage, which holds cultural significance and is believed to enhance neonatal well-being. Despite its prevalence, empirical evidence supporting the efficacy of oil massage remains limited. This study, conducted in a rural setting, aims to bridge traditional practices with evidence-based care, exploring the impact of oil massage on newborn behavioural responses. A quasi-experimental design involving 60 newborns (30 in each group) assessed behavioural responses through a pre and post-test approach. Results indicate a significant improvement in selected behavioural responses among newborns receiving oil massage, emphasizing its potential integration into routine care. The control group showed a pre-test mean of 14.83 (SD = 2.41) and a post-test mean of 16.23, while the experimental group exhibited a pre-test mean of 15.83 (SD = 1.80) and a post-test mean of 26.07. T-test values of 5.194 for the control group and 26.137 for the experimental group were indicative of statistically significant changes. The study contributes insights into neonatal care practices, urging further exploration of contextual intricacies and demographic influences on newborn behaviour.

## Background:

During the initial weeks of a newborn's life, rapid physiological and behavioural adjustments take place. The infant undergoes significant developmental changes, such as adapting to breathing outside the womb, regulating body temperature, and establishing feeding patterns. Sleep-wake cycles begin to develop, and the baby starts to respond to stimuli, forming the foundation for future cognitive and sensory development. This critical period requires attentive care and support to ensure the healthy growth and well-being of the newborn [1]. In many cultural contexts, oil massage is a time-honoured practice believed to confer various benefits to the neonate [2]. This traditional approach is thought to promote bonding between the caregiver and the newborn, aid in relaxation, and contribute to the baby's overall well-being by enhancing skin health and circulation. The practice often serves as a cherished ritual passed down through generations[3]. Despite its prevalence, the empirical evidence supporting the efficacy of oil massage in promoting selected behavioural responses remains limited[4]. While the practice is culturally significant and valued for bonding, relaxation, and skin benefits, further research is needed to establish its specific impacts on newborn behaviour based on scientific evidence [5]. In the tapestry of neonatal care, practices such as oil massage have persisted across generations, shaped by cultural beliefs and familial customs. While these practices are deeply embedded in the fabric of daily life, their validation through systematic inquiry is essential to discern their tangible effects [6]. This study recognizes the need to bridge traditional practices with evidence-based care, particularly in the context of newborn well-being. The rural setting adds a distinctive dimension to our investigation. Acknowledging potential disparities in healthcare practices and accessibility, we aim to unravel the intricacies of oil massage's impact within this demographic. The socio-cultural and economic nuances of rural life can significantly influence health-related practices, necessitating a focused exploration of the efficacy of oil massage in promoting positive behavioural responses among newborns in this setting [7, 8]. Understanding the behavioural responses of newborns is pivotal, serving as a barometer for their overall well-being and adaptability to their external environment [9]. Therefore, we are interested in assessing and comparing behavioural responses between two groups: newborns subjected to daily oil massage [experimental group] and those without the intervention (control group).

## Methodology:

## Study design:

A quasi-experimental pre and post-test design was employed to assess the impact of oil massage on selected behavioural responses among normal newborns.

## Study Setting and participant:

The study was conducted in a rural area of Visnagar, chosen to capture the unique dynamics and healthcare practices prevalent in rural settings. Sixty newborns were included in the study, with 30 assigned to the experimental group and 30 to the control group.

## Sampling Technique:

Anon-probability convenient sampling method was chosen for the current research [10].

## Inclusion criteria:

[1] Full-term and normal-weight newborns without any known medical complications

[2] Newborns with caregivers willing to participate in the study

## Exclusion criteria:

[1] Newborns with congenital abnormalities or significant health issues

[2] Caregivers unwilling or unable to adhere to the study protocol

## Procedure:

Caregivers of the experimental group were instructed to administer oil massage to their newborns once a day for duration of 15 days, following a standardized protocol. The control group received routine care without the inclusion of oil massage during the study period.

## Data collection:

Pre and post-tests were conducted for both experimental and control groups to assess selected behavioural responses. Behavioural responses were measured using a validated assessment tool designed for neonatal behavioural evaluation. Demographic information, including age, gender, religious background, mother's education, mode of delivery, birth weight, frequency of sleep, sleep duration, and frequency of feeding, was collected.

## Data analysis:

Descriptive statistics, including mean and standard deviation, were calculated for demographic variables and selected behavioural responses. The independent t-test was employed to compare pre and post-test results between the experimental and control groups. Chi-square tests were used to explore associations between demographic variables and behavioural responses.

## Ethical considerations:

The study adhered to ethical guidelines, obtaining informed consent from caregivers before participation. Participants were assured of confidentiality, and their right to withdraw from the study at any point was respected.

## Results:

The distribution of participants according to demographic characteristics demonstrates a balanced representation in both the control and experimental groups (Table 1). Notably, the age distribution shows a relatively equal spread across the three intervals (7-14 days, 15-21 days, and 22-28 days) in both groups, with the majority falling within the 7-14 days category. The gender distribution is also evenly divided, with an equal number of male and female participants in both groups. Regarding religious backgrounds, the majority in both groups belong to the Hindu faith. In terms of maternal education, the distribution is fairly consistent, with a significant proportion having completed education up to the 1-10th standard. The mode of delivery, birth weight, frequency of sleep, sleep duration, and frequency of feeding also demonstrate varied yet comparable distributions across both groups.

The chi-square analysis of demographic variables reveals several noteworthy trends. For age in days, gender, religion, mother's education, mode of delivery, birth weight, frequency of sleep, sleep duration, and frequency of feeding, the chi-square values indicate non-significant associations, suggesting that these demographic factors are not significantly different between the experimental and control groups. However, a significant association is observed in the frequency of feeding, indicating that this behavioural response is influenced by demographic variables. In the control group, the pre-test mean stood at 14.83 (SD = 2.41), experiencing an increase to 16.23 in the post-test. Conversely, the experimental group exhibited a slightly higher pre-test mean of 15.83 (SD = 1.80) and demonstrated a substantial rise to 26.07 in the post-test. T-test values of 5.194 for the control group and 26.137 for the experimental group were indicative of statistically significant changes. With degrees of freedom set at 29 for both groups, the critical value from the t-distribution table (two-tailed, α = 0.05) was determined to be 2.04.

## Discussion:

The major finding of this study reveals a significant improvement in selected behavioural responses among normal newborns following oil massage. This aligns with similar research, such as the study by Jabraeile *et al.* (2016), affirming the positive impact of oil massage on newborn behaviour [11]. Another study conducted by Rebecca *et al.* (2011) also suggested a significant effect of oil massage on selected behavioural responses [12]. The observed positive changes in behavioural responses echo findings from studies like Priyadarshi *et al.* (2022), reinforcing the therapeutic value of oil massage for newborns [13]. However, it's crucial to acknowledge variations in findings across studies. For instance, the study by Andrew Vickers *et al.* (2001) suggested a less pronounced impact of oil massage, emphasizing the need for further investigation into variations in massage techniques, oils used, and cultural considerations [14]. These differences highlight the complexity of neonatal responses to oil massage and the importance of considering contextual factors. The identified significant association between feeding frequency and selected behavioural responses adds a unique contribution to the study. This aligns with the work of Griffith *et al.* (2017), who similarly found demographic influences on feeding patterns [15, 16]. However, the non-significant associations with other demographic variables differ from the findings of Kelmanson *et al.* (2006), who reported significant correlations with sleep patterns and birth weight. These nuanced differences underscore the complex interplay of demographic factors in influencing newborn behaviour, requiring more focused investigation. Comparative analysis of these major findings with existing studies emphasizes both commonalities and divergences. While the present study aligns with Kulkarni *et al.* (2010) positive outcomes, variations with Gary L Darmstadt and Saha *et al.* (2002) highlight the complexity of neonatal responses to oil massage. The consistency in positive changes in behavioural responses across studies, however, emphasizes the robustness of the observed effects. The effectiveness of oil massage in enhancing selected behavioural responses, as demonstrated in the present study, is a major breakthrough. The substantial improvements align with the findings of studies like *et al.* (2015) [17], reinforcing the therapeutic value of oil massage for newborns. Despite differences, the overall consensus supports the positive influence of oil massage on newborn behaviour. Comparative analysis with existing studies underscores both commonalities and discrepancies. While the present study aligns with certain positive outcomes, variations with other studies emphasize the multifactorial nature of neonatal responses to oil massage. These differences may be attributed to variations in cultural practices, regional differences, and methodological nuances in study designs.

## Implications and Recommendations:

The significant improvement in selected behavioural responses among newborns following oil massage has significant implications for neonatal care practices. The positive impact suggests that incorporating oil massage into routine care protocols could contribute to enhanced newborn well-being. Healthcare professionals and caregivers should be informed about the potential benefits of this traditional practice. However, the study's divergent results with certain demographic variables indicate that a one-size-fits-all approach may not be suitable. Therefore, further research, possibly through qualitative methods, is recommended to explore the contextual nuances influencing the efficacy of oil massage in different settings. The association between feeding frequency and selected behavioural responses highlights the interconnectedness of various caregiving practices. Interventions focusing on both oil massage and feeding routines may have a synergistic effect, warranting further exploration in future studies. Additionally, understanding the influence of demographic variables on neonatal behaviour is essential for tailoring interventions to specific population needs.

## Conclusion:

Data provides insights into the impact of oil massage on newborn behavioural responses, emphasizing both the positive outcomes and the need for nuanced interpretations. The observed improvements align with existing literature, supporting the integration of oil massage into neonatal care practices. However, the variations in results across demographic variables underscore the complexity of neonatal behaviour. Future research should delve into the contextual intricacies influencing the effectiveness of oil massage, considering regional, cultural, and individual differences. This will contribute to the refinement of evidence-based practices in neonatal care, ensuring tailored approaches that address the diverse needs of newborns in different settings.

## Figures and Tables

**Figure 1:**
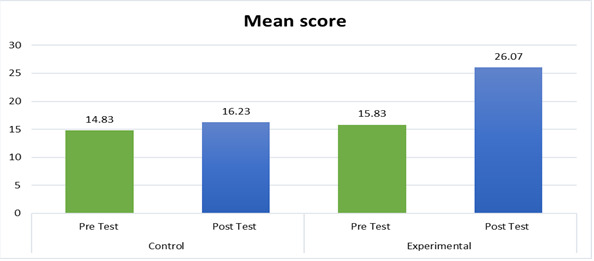
mean distribution according to pre-test and post test result in control and experimental group

**Table 1:** Frequency and percentage distribution of experimental and control groups according to demographic variables

**S. No.**	**Characteristics**	**Control group**		**Experimental group**	
		**F**	**%**	**F**	**%**
1	Age in days				
	14-Jul	9	30.00%	14	46.70%
	15-21	13	43.30%	11	36.70%
	22-28	8	26.70%	5	16.70%
2	Gender				
	Male	15	50%	17	50%
	Female	15	50%	13	50%
3	Religious				
	Hindu	22	73.30%	26	86.70%
	Muslim	6	20.00%	4	13.30%
	Other	2	6.70%	-	-
4	Mother's education				
	Illiterate	14	46.70%	12	40.00%
	1-10th pass	15	50%	15	50%
	12th pass or graduate	1	3.30%	3	10.00%
5	Mode of delivery				
	Normal vaginal delivery	17	56.70%	13	43.30%
	L.s.c.s	15	50%	15	50.00%
6	Birth weight in grams				
	2500-3000	22	73.30%	13	100%
	3001-3500	7	23.30%	12	40.00%
	3501 and above	1	3.30%	5	16.70%
7	Frequency of sleep				
	4-5 times	15	50.00%	13	43.30%
	6-7 times	13	43.30%	15	50%
	8-9%	2	6.70%	2	6.70%
8	Sleep duration				
	Less than 1 hour	15	50%	12	40%
	1-2 hour	14	46.70%	16	53.30%
	More than 2 hour	1	3.30%	2	6.70%
9	Frequency of feeding				
	Less than 6 times/day	14	46.70%	12	40.00%
	7-8 times/day	15	50.00%	16	53.30%
	More than 9 times/day	1	3.30%	2	6.70%

**Table 2:** Data association between demographic variables in experimental and control groups

**Demographic Variable**	**Control group Chi Square**	**Experimental group Chi Square**	**Table Value**	**DF**
Age in Days	1.102NS	0.723	5.99	2
Gender	0.001 NS	1.33 NS	3.84	1
Religion	0.779 NS	0.923 NS	5.99	2
Mother Education	0.077 NS	1.32 NS	5.99	2
Mode of Delivery	2.802 NS	0.24 NS	3.84	1
Birth Weight (grams)	0.779 NS	1.615 NS	5.99	2
Frequency of Sleep	2.802 NS	0.535 NS	5.99	2
Sleep Duration	2.143 NS	4.05 NS	5.99	2
Frequency of Feeding	0.077 NS	11.25*	5.99	2
NS= Non significant, *= significant
